# Sprengel’s deformity of the shoulder: Current perspectives in management

**DOI:** 10.4103/0973-6042.80459

**Published:** 2011

**Authors:** Aditya Sai Kadavkolan, Deepak N. Bhatia, Bibhas DasGupta, Pradeep B. Bhosale

**Affiliations:** Department of Orthopaedic Surgery, Seth GS Medical College & King Edward VII Memorial Hospital, Parel, Mumbai, India

**Keywords:** Congenital anomaly, shoulder, Sprengel’s deformity

## Abstract

Sprengel’s deformity or congenital elevation of scapula is a complex deformity of the pectoral girdle, and results in symptomatic cosmetic and functional disability. Several studies have attempted to analyze the three-dimensional aspects of this deformity; optimal methodologies of quantification and surgical correction techniques have been debated since the condition was first described. This article presents a concise review of the exact pathoanatomy, clinical presentation, imaging techniques, and surgical procedures described in the management of this condition.

## INTRODUCTION

Congenital elevation of the scapula or ‘Sprengel’s shoulder’ is an anomaly of the shoulder girdle that is associated with abnormal descent, and altered position and anatomy of the scapula. The deformity is usually associated with muscle hypoplasia or atrophy, and a combination of these factors results in disfigurement and functional limitation of the shoulder.[1] The earliest description of this condition was reported in 1863 by Eulenberg; he reported three cases of *hochgradige dislocation der scapula* and described its association with dorsal scoliosis. Subsequent cases were described in 1880, 1883, and a method of surgical correction was suggested in 1888.[2] In 1891, Sprengel drew attention to this deformity, which he described as *angeborene verschiebung des schulterblattesnachoben*, and advocated a plausible theory for the existence of this deformity.

## DEVELOPMENTAL, NORMAL AND PATHOLOGICAL ANATOMY

The scapula develops embryologically along with the upper limb; it appears during the fifth week in the upper dorsal / lower cervical region with the arm bud and descends up to the final anatomical position of one of the second-to-eighth dorsal vertebrae by 12 weeks of gestation.[2–5] The horizontal diameter, measured at the base of the spine of the scapula, is less than the vertical diameter. Broca suggested a “scapular index” to describe the relationship between the horizontal and the vertical lengths of the scapula; this was represented by a formula (100× breadth/length) and calculated to be approximately between 63 and 71.[2] Similarly, others have used a “subscapular angle,” a “supraspinous angle” and an “infraspinous angle” to describe deviation from the normal.[2]

The trapezius inserts along the medial border of the scapula and resists the upward-directed forces of the levator scapulae and the rhomboids; it has been reported to be atrophied in cases of acquired elevation of the scapula.[2] The other muscles acting on the scapula are pectoralis major, rhomboids, levator scapulae, serratus anterior and latissimus dorsi; varying degrees of involvement of these muscles may be seen in congenital elevation of the scapula.

The pathology in Sprengel’s deformity probably represents a continuance of the fetal form of the scapula. The smaller deformed scapula has a horizontal diameter that exceeds the vertical diameter; anterior curving of the supraspinous portion with prolongation of the superior medial scapular angle has been described. Other pathoanatomical findings include an omovertebral bar, articulations with the vertebral column; and these may extend from the superomedial scapular angle or the upper third of the medial border of the scapula up to the transverse process of a cervical vertebra (one of the fourth-to-seventh vertebrae).[1,2,4–11] Additionally, costovertebral defects (spina bifida and kyphoscoliosis) and underdevelopment of pectoral girdle bones (clavicle, humerus) and musculature (pectoralis major, trapezius) may coexist.[2,4,7]

## CLINICAL FEATURES

**Figure 1:**
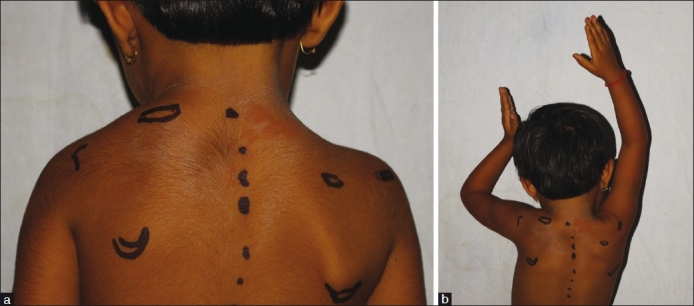
(a) Cosmetic aspect of Sprengel’s deformity is shown. The landmarks show marked elevation of the left scapula as compared with the right; (b) Functional aspect of Sprengel’s deformity is shown. Marked restriction of abduction on the left side is seen as compared to the right

The demographics, clinical presentation, common clinical associations, and differential diagnoses are summarized in Tables 1–4. The cosmetic aspect of the deformity has been classified by Cavendish into four grades in an attempt to simplify indications for treatment [Table 5].[9] The functional aspect of the deformity has been attributed to (a) a forward curvature of the superior angle of the scapula over the apex of the thorax, (b) abutment of the medial scapular border against the spinous processes of adjacent vertebrae and (c) the omovertebral bone [Figure 1a–b].[10] Syndromes associated with Sprengel’s deformity include teratological conditions such as inencephaly (a triad of occipital defect, spina bifida of cervical vertebrae, and fixed retroflexion of the head) and the Klippel-Feil syndrome [Table 6].[8]

**Table 1:** Clinical presentation of congenital elevation of the scapula

Cosmetic
High position of the scapula
Scoliosis
Torticollis
Caput obstiosum (asymmetric distortion of the skull)
Facial asymmetry
Functional
Restricted motions of scapula and scapulo-humeral joint

**Table 2:** Demographics of Sprengel’s deformity

Age – Mostly noticed at birth
Gender – Equal distribution in both sexes
Side – Left side more common than right, bilateral only in 10&
Hereditary – May be associated with other congenital anomalies in the family

**Table 3:** Differential diagnosis of congenital elevation of the scapula

Rickets
Osteomalacia
Malunited scapular fractures
Paralysis
Cervical tuberculosis

**Table 4:** Demographics of Sprengel’s deformity

Talipes equino-varus
Pes valgus
Hallux valgus
Shortening of femur
Shortening of leg and foot, congenital dislocation of the hip
Defect of hand or fingers
Radial defect
Dislocation of radial head, maldevelopment of the whole upper extremity
Bifid ear
Cleft palate
Extremely arched palate
Adenoid
Strabismus
Underdevelopment of mammary on side opposite to elevation
Supernumerary mammary gland on side of affected scapula
Subcostal tumor
Dextrocardia
Floating kidney
Congenital inguinal hernia
Left congenital inguinal hernia
Anal ectopy with incomplete atresia
Inencephaly

**Table 5:** Cavendish classification

Grade I (Very mild)	Shoulders level; deformity invisible when patient is dressed
Grade II (Mild)	Shoulders almost level; deformity visible as a lump in the web of the neck when patient is dressed
Grade III (Moderate)	Shoulder joint is elevated 2-5 centimeters; deformity visible
Grade IV (Severe)	Shoulder joint is elevated; superior angle of the scapula near the occiput

**Table 6:** Klippel-Feil syndrome and Sprengel’s deformity

Congenital fusion of at least 2 cervical vertebrae with/without additional spinal/extraspinal manifestations
Associated Sprengel’s deformity: 7%-42%
Most common congenitally fused segment in Sprengel’s deformity: C6-C7; extensive fusion patterns common
Thorough neurological examination to be done preoperatively to avoid complications during surgery and anesthesia

## IMAGING

**Figure 2:**
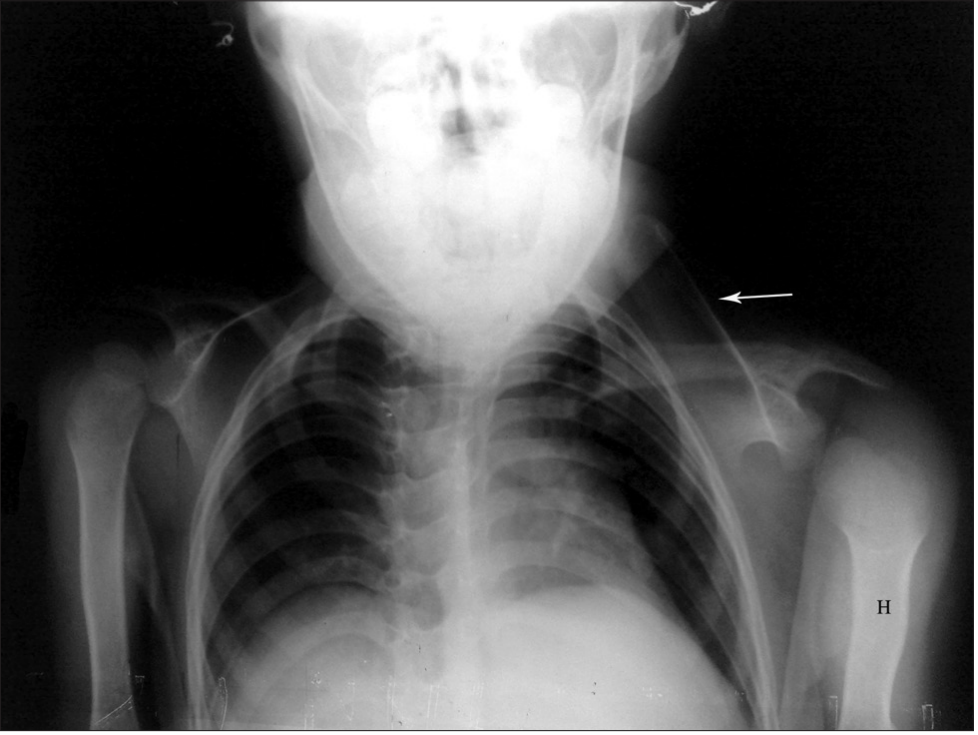
Oblique radiograph (arrow – omovertebral bar; H – Hypoplastic humerus)

**Figure 3:**
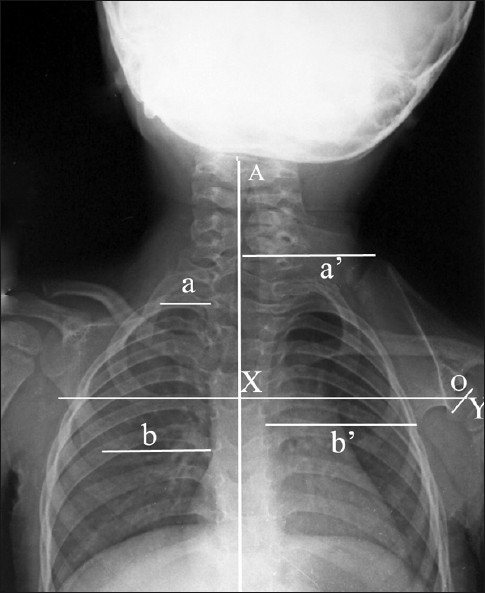
Frontal radiograph of a patient with Sprengel’s deformity (Rigault grade III). (X – Line drawn perpendicular to the body axis ‘A’; Y – Line joining the superior and inferior edges of the glenoid; O – Angle between lines X and Y; b: distance between the inferior angle of the normal scapula and the spine; b’ – Distance between the inferior angle of the affected scapula and the spine; a – The distance between the superior angle of the normal scapula and the spine; a’ – Distance between the superior angle of the affected scapula and the spine

**Figure 4:**
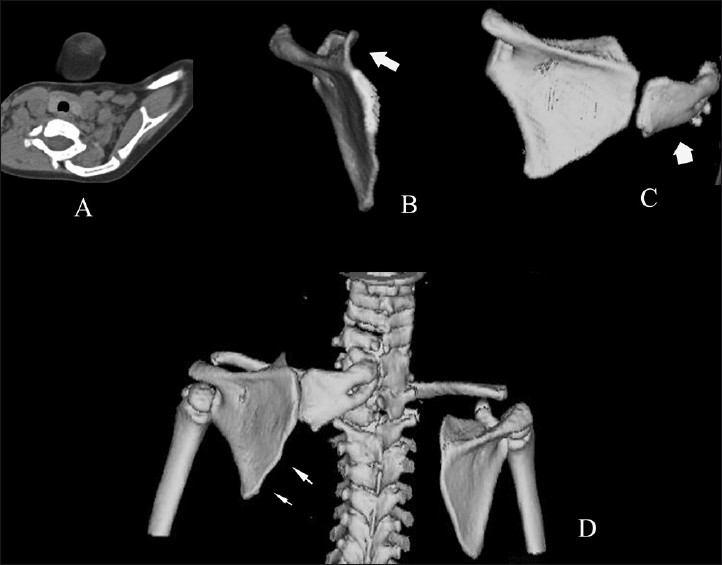
CT scan (A) and 3D reconstruction (B, C and D) show the omovertebral connection (thick arrow) arising from the medial border of the scapula and the vertebral column. (B) shows anterior curving of the supraspinous portion of the affected scapula (arrow). (D) shows the convex medial border and the concave lateral border of the affected scapula (multiple arrows)

**Figure 5:**
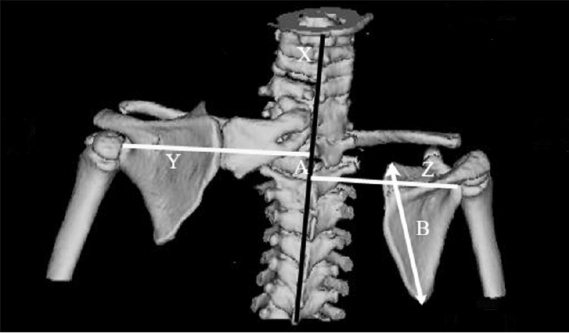
Superior displacement of the affected scapula is represented as a ratio of A to B. Y represents a line drawn from the center of the glenoid of the abnormal scapula, Z represents a line drawn from the center of the glenoid of the right scapula, both Y and Z are perpendicular to the vertebral axis, and X is the vertical body axis; A is the distance between the points at which the lines intersect the spinal column; B is the vertical height of the normal scapula

**Figure 6:**
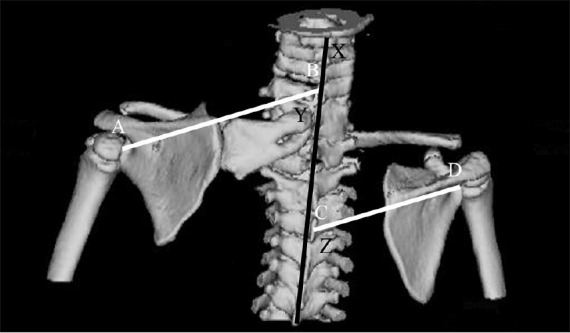
Rotational deformity of the affected scapula is represented by the difference between lines Z and Y (X – Vertebral column axis; AB – A line joining the center of the glenoid and the base of scapular spine to the vertebral column axis on the affected side; CD – A line joining the center of the glenoid and the base of scapular spine to the vertebral column on the normal side; Y – Angle between AB and X; Z – angle between CD and X)

Plain radiographs can be used for assessing the deformity and presence of omovertebral communication, and to note the postoperative correction. Based upon the frontal radiographs and the relation of the superomedial angle to the vertebral column, Rigault proposed a classification to assess the deformity [Table 7].[12] Woodward described an oblique view to assess the presence of the omovertebral bone between the superomedial angle of the scapula and the vertebral column [Figure 2].[10] Ahmad described the distances between the inferior and the superomedial angles of the scapula and the spinal column, and the angle of glenoid tilt, to assess the postoperative outcomes [Figure 3].[13] Cho *et al*. used three-dimensional CT (3D-CT) to evaluate scapular dysplasia and malpositioning and suggested roles in preoperative planning. These authors described the affected scapulae to be larger than the contralateral ones, and demonstrated a decrease in their height-to-width ratio without a significant difference in glenoid version. Moreover, they found an inverse relationship between scapular rotation and superior displacement [Figures 4–6].[1].

**Table 7:** Rigault’s classification

Grade 1: Superomedial angle lower than T2 but above T4 transverse process
Grade 2: Superomedial angle located between C5 and T2 Transverse process
Grade 3: Superomedial angle above C5 transverse process

## TREATMENT

The goal of surgical intervention for Sprengel’s deformity is cosmetic and functional improvement; however, the condition is often associated with other anomalies such as torticollis and congenital scoliosis, which limit the amount of correction that can be obtained. Patients with bilateral deformities or grade 1 Cavendish deformity may be observed for progression; patients with grade 4 deformities should be offered a guarded prognosis; and surgery may be avoided in patients with short necks and concomitant grade 4 deformity.[9] Surgical intervention before the age of 2 years is extensive and is technically more difficult. Best results are obtained if surgery is performed below the age of 5 years.[7,14] Jeannopoulos advised a resection of the superomedial part with omovertebral bone excision as the only procedure in patients above the age of 6 years.[7] Factors to be assessed while evaluating a patient for surgery include severity of the deformity, functional impairment, age, and associated comorbid conditions.[10] Hence surgery is best advisable for a patient between 3 and 8 years of age, and with moderate or severe cosmetic/ functional deformity. The presence of associated congenital anomalies may be contraindications to operation.[10]

**Figure 7:**
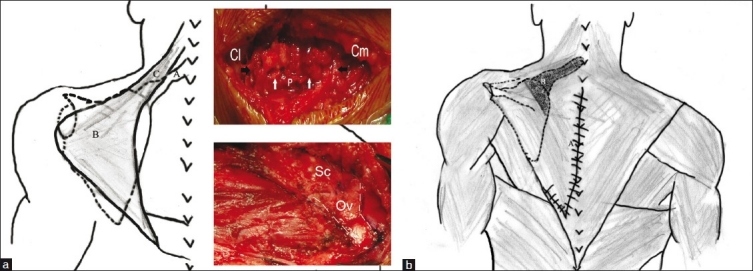
(a) Diagrammatic representation of Woodward’s procedure: The origins of trapezius and rhomboids (B) are resected from the spinous processes (A – Omovertebral bar; B – Elevation of trapezius and other scapular musculature; C – Levator scapulae). Inset – Top right shows morselization of the clavicle; ‘Cl’ and ‘Cm’ – The lateral end and the medial end of the clavicle, respectively; P – Periosteum sutured over the morselized part; Black arrows – Extent of the morselization; White arrows – Sutured periosteum.; (b) Shaded area indicates extraperiosteal excision of the omovertebral bar and the supraspinous portion of the scapula. The aponeurosis of trapezius and rhomboids (A) are sutured over the scapula in the corrected position

**Figure 8:**
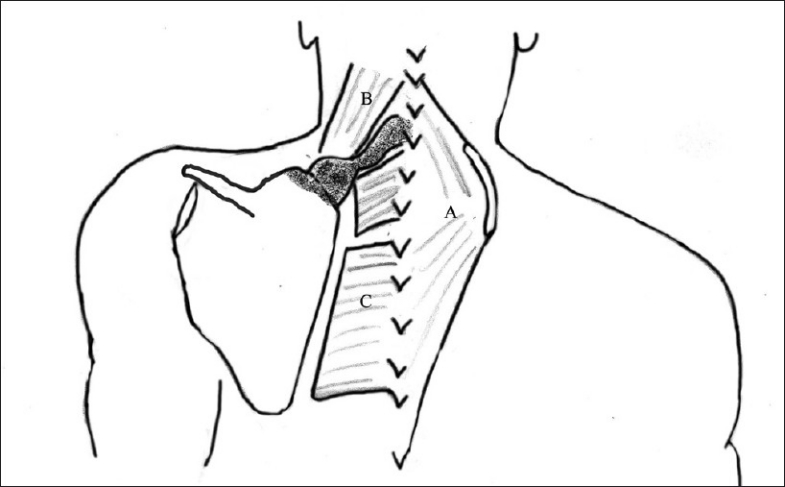
Diagrammatic representation of Green’s procedure. Shaded region indicates the area of resection (omovertebral bar, supraspinous fossa). (A – Dis-inserted trapezius; B – Levator scapula; C – Dis-inserted rhomboids – major and minor)

The surgical procedures involve a combination of (a) scapular lowering with either the shift of the origin or the insertion of the scapular muscles on the spine/ scapula, (b) resection of the superomedial border and (c) omovertebral bar resection[9,15] [Table 8]. A clavicular morselization is sometimes recommended as a concomitant deformity of this bone may reduce the correction obtained.[9] Putti’s procedure consisted of detachment of the scapular insertion of the rhomboids and trapezius, omovertebral bar resection, followed by lowering the scapula and fixing its inferior angle to a rib at the corrected level.[7] Shrock modified Putti’s procedure by suggesting subperiosteal dissection of the musculature and adding an osteotomy of the supraspinous scapular region and the acromial base to facilitate scapular descent.[5] Woodward described a procedure for correction of the deformity, involving distal shift of the origin of the trapezius and the rhomboids on the spine [Figure 7a–b]. This was maintained by placing the scapula in a pocket of the trapezius muscle. Ahmad proposed a modification to the Woodward’s procedure, for achieving better abduction and correction of the glenoid tilt; the scapula was anchored to the lower dorsal vertebrae by a stout absorbable suture placed through the superomedial scapula, so as to externally rotate it and cause lateral displacement of the inferior angle, thereby achieving correction of glenoid vara.[16] Green described a technique that involved resection of the prominent superior scapular border and extra-periosteal division of the muscular attachments of scapula to allow the scapula to be displaced inferiorly and muscular reattachment at the newer corrected level at the scapula [Figure 8]. Andrault *et al*. suggested modifications to Green’s procedure; these included (a) dis-insertion of supraspinatus, (b) clavicular osteotomy and (c) a limited release of the serratus anterior to facilitate the descent of the scapula. The authors suggested that the incidence of brachial plexus palsy could be reduced by clavicular osteotomy, and that scapular winging could be prevented by doing only a limited release of serratus anterior from the medial scapular border.[17]

**Table 8:** Surgical procedures for Sprengel’s deformity

Procedure	Incision	Muscular detachment	Scapular osteotomy	Omovertebral bar excision	Remarks
Shrock’s modification of Putti’s procedure[5]	Paramedian	Both muscles inserting on the medial border as well as lateral border; detached subperiosteally	Supraspinous fossa osteotomy	Yes	Acromion base osteotomy
Woodward’s scapular transplantation procedure[11]	Midline	At the muscular origin at the spinal column	Yes, if excess curving of the supraspinous region	Yes	
Wilkinson’s osteotomy / Vertical scapular osteotomy[19]	Paramedian	At the insertion along the medial border and the spine of scapula	Vertical osteotomy	Yes	
Green’s procedure±modifications[11,16]	Midline	At muscular insertion at the scapula	Supraspinous fossa osteotomy	Yes	
Mears’ procedure[15,18]	Midline	At the muscular insertion at the scapula	Superolateral border osteotomy, supraspinous fossa osteotomy	Yes	Detachment of the triceps to gain abduction
Partial scapulaectomy[20]	Inverted L-shaped	The muscles inserting along medial border are sharply detached; subperiosteal elevation of supraspinatus and infraspinatus	Superior portion of the spine of scapula at its midpoint	Yes	

**Figure 9:**
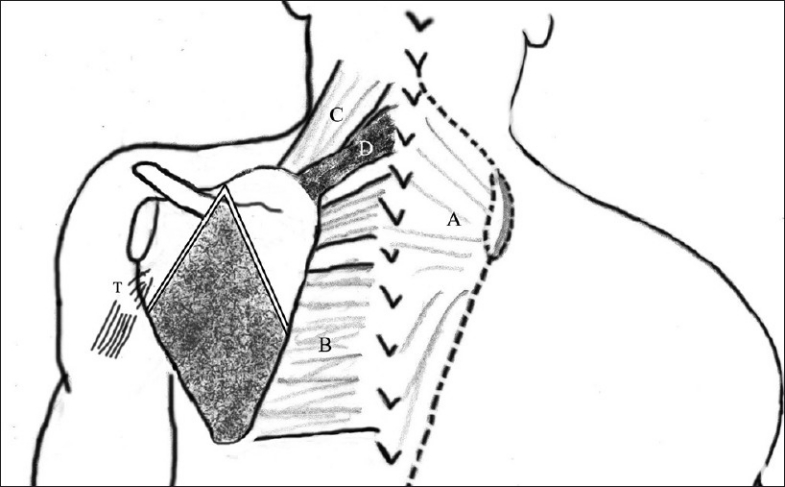
Diagrammatic representation of Mears’ procedure. The shaded region represents the area to be osteotomized (A – Reflected trapezius; B – Rhomboids; C – Levator scapulae; T – The detached triceps)

Mears described a procedure which involved (a) subperiosteal elevation of the scapular musculature, (b) extraperiosteal resection of the omovertebral bone, (c) supraspinatous fossa osteotomy, (d) release of long head of triceps and a portion of the origin of teres minor from the scapula and (e) resection of the superolateral border of the scapula to gain abduction [Figure 9]. The gain in shoulder abduction is more with this procedure as compared to the others described for Sprengel’s deformity.[15,18] Other procedures described include vertical scapular osteotomy and partial scapulaectomy. Wilkinson *et al*. described a vertical scapular osteotomy which consisted of a vertical osteotomy, 1 cm from the vertebral edge of the scapula so as to lower the lateral part till both the spines were at the same level.[19] Zhang *et al*. described a partial scapulaectomy for correction of the scapular deformity; the superior potion of the spine of scapula was osteotomized, and the omovertebral bar was excised. The adequacy of the correction was judged by comparing the superior borders of the two scapulae.[20]

## RESULTS

A number of procedures have been described for correction of the Sprengel’s deformity, and an equally large number of studies have been attempted to assess the outcome [Table 9]. A direct comparison of the results is difficult as various authors have used different techniques for correction and different outcome-assessment measures. The inferior angle, the superior border, the spine or the center of the scapula, all have been used to assess the correction.[10,11,20,21] Gain in range of abduction is comparable and its mean value ranges from 19° to 70° in various studies, with a greater gain seen in patients with a more severe preoperative restriction.[9–11,17–25] Surgical complications described include scar-related complications, brachial plexus palsy, brachial neuritis, winging of the scapula, regeneration of the omovertebral bar, recurrence of the deformity and prominence of the sternoclavicular joint.[9–11,15–23] Current recommendation from studies analyzed include the following: a clavicular osteotomy may be added in severe cases to facilitate scapular descent without causing neurovascular complications[2]; Woodward’s procedure may be avoided in patients with pre-existing scapular winging[3]; there is no constant correlation of anatomical and cosmetic results with recurrence of the deformity, age of the patient and functional outcome.[7,10,17,21]

**Table 9:** Results of surgical procedures for Sprengel deformity

Authors	Procedure	Follow-up period	n	Results	Complications	Remarks
Jeannopoulos C L (1926)[7]	Shrock procedure	7 y (range, 1-17 y)	16**	Cosmesis: good (7); fair (4); no improvement (5). Abduction: ‘excellent’ range obtained in 5 of 7 patients with initially restricted range	Recurrence (6); exostosis regeneration (9); keloid (7); winging of the scapula (6); sternoclavicular joint prominence (2); BPP (2)	
Woodward J W (1961)[11]	Woodward scapular transplantation	2.5 y (range, 9-60 months)	9	H: mean, 5.2 cm (range, 4-8 cm) A: mean, 35.5°(range, 20°-70°)	Scar (3); transient BPP (1)	Scapular spine to judge correction
Carson *et al*. (1981)[10]	Woodward scapular transplantation	5.7 y (range, 2.5-11 y)	11; 8 available for follow-up	H: mean, 1.6 cm (range, 0.3-4.3 cm) A: mean, 50°(range, 35°-60°), in patients with severe preoperative restriction of abduction (n=5); overall mean A: 29° Cavendish gr. 1 outcome (6) Cavendish grade 2 outcome (2)	Scar (7); scapular winging (1)	Subcuticular sutures to decrease the scar-related complications; inferior angle of the scapula to judge correction
Grogan *et al*. (1983)[21]	Woodward scapular transplantation	8 y 9 mo (range, 3 mo to 17 y)	20 patients, 21 scapulae; 13 patients for follow-up	H: mean, 2 cm (range, 0-3.7 cm); A: mean, 37°(range, 5°-85°)	Transient BPP (1); scar (1); exostosis regrowth (1); exaggeration of winging of scapula (1)	Clavicular osteotomy to gain more correction with less risk of neurovascular compression; center of the scapula to judge correction
Cavendish M E (1972)[9]	Woodward scapular transplantation	Not reported	5	Improved function (4) Cavendish grade 1 outcome (1); grade 3 outcome (3)	Scar (1)	−
Cavendish M E (1972)[9]	Excision of the omovertebral bar and superomedial scapula	Not reported	18	Cavendish grade 1 outcome (10); Cavendish grade 2 outcome (5); Cavendish grade 3 outcome (3). Functionimproved (8); same (7); worse (3)	Scar (5)	
Cavendish M E (1972)[9]	Subtotal scapulaectomy	Not reported	7	Cavendish grade 2 outcome (6); Cavendish grade 3 outcome (1)	Scar (3)	Worst scarring, poor function and intraoperative bleeding
A A Ahmad (2010)[16]	Modified Woodward’s procedure	36.2 mo (range, 24-51 mo)	15 shoulders, 11 patients	A: 49°Cavendish grade 1 outcome (7); Cavendish grade 2 outcome (8)	Winging of the scapula (4); keloid (4)	Increased postoperative range of abduction compared to Woodward’s procedure
Leibovic *et al*. (1990)[22]	Green’s procedure	15 y	16*** shoulders, 14 patients; 15 shoulders in follow-up	H: mean, 1.7 cm; A: mean, 57°(range, 20°-90°)	Winging of scapula (2); hypertrophic scars (6); no neurovascular injuries	Place scapula in a pocket of latisimus dorsi
Mears D C (2001)[15]	Mears’ procedure	5.5 y (range, 3-15 y)	8	Flexion improved to 175°(range, 170°- 180°); abduction to 150°(range, 120°- 170°)	Scar/ keloid (2); exostosis (1)	Removal of the long head of triceps to enhance the glenohumeral range of abduction
Masquijo *et al*. (2009)[18]	Mears’ procedure	5 y (range, 1-6 y)	14	F: mean, 70°(range, 50°-110°); A: mean, 64°(range, 10°-80°). Improvement by average 2 grades (Cavendish)	Exostosis (2); keloids (2)	
Wilkinson *et al*. (1980)[19]	Vertical osteotomy	4.5 y (range, 1-10 y)	12	A: mean, 54°(range, 15°-85°). Cavendish grade 1 outcome (6); grades 2-3 outcomes (5)	Prominence of the inferior angle of scapula (1); brachial neuritis (1)	Clavicular osteotomy to facilitate descent may be added.
McMurtry *et al*. (2005)[23]	Vertical osteotomy	10.4 y (range, 1-17 y)	12	A: mean, 53°(range, 30°-60°) † Cavendish grade 1 outcome (7); grade 2 (4); grade 3 (1)	BPP (1)	Resect at least 50% of the body to gain the range of abduction. To be avoided in children with brevicollis
Zhang *et al*.[20]	Partial scapulaectomy	Study duration: 9 y	26 (28 shoulders)	Two groups: Group A (preoperative abduction >120°); A: mean, 52.22°±15.01 Group B (preoperative abduction <120°); A: mean, 19°±17.28 Cosmetic improvement in 82%	Winging of the scapula (1)	Aimed to achieve function over cosmesis; scapula not brought to the same level of the inferior angle of the contralateral side

BPP – Brachial plexus palsy; H – Scapular lowering obtained; A – Improvement in abduction; F – Improvement in flexion; E – Antepulsion improvement; y – Years; *n* – The number of patients included in the study;

** Two groups were described in the study: Only subperiosteal stripping with superomedial scapular resection without correcting position was done in 4 patients; Scapular position was corrected in 16 patients; the results were poor in the former group, except 1 patient, in whom spontaneous correction was obtained;

*** The study cohort was of 18 shoulders, 16 patients, but 2 patients underwent limited procedures due to their age and are excluded here;

† One patient had a recurrence of Erb’s palsy, and there was loss of abduction — this patient is not included

## CONCLUSIONS

Sprengel’s deformity of the shoulder is a dysplasia of the pectoral girdle, resulting in cosmetic and functional disability. The deformity is associated with other congenital anomalies, which often dictate the management and outcome of treatment. Surgical techniques described in literature provide a satisfactory cosmetic and functional outcome, and a low complication rate.
